# Engineering the *Escherichia coli* Nitroreductase NfsA to Create a Flexible Enzyme-Prodrug Activation System

**DOI:** 10.3389/fphar.2021.701456

**Published:** 2021-06-07

**Authors:** Abigail V. Sharrock, Sarah P. McManaway, Michelle H. Rich, Jeff S. Mumm, Ian F. Hermans, Moana Tercel, Frederik B. Pruijn, David F. Ackerley

**Affiliations:** ^1^ School of Biological Sciences, Victoria University of Wellington, Wellington, New Zealand; ^2^ Centre for Biodiscovery, Victoria University of Wellington, Wellington, New Zealand; ^3^ Maurice Wilkins Centre for Molecular Biodiscovery, Auckland, New Zealand; ^4^ Auckland Cancer Society Research Centre, The University of Auckland, Auckland, New Zealand; ^5^ The Wilmer Eye Institute, Johns Hopkins University, Baltimore, MD, United States; ^6^ Malaghan Institute of Medical Research, Wellington, New Zealand

**Keywords:** nitroreductase (NTR), bystander effect, targeted cell ablation, enzyme-prodrug therapy, GDEPT, BDEPT

## Abstract

Bacterial nitroreductase enzymes that can efficiently convert nitroaromatic prodrugs to a cytotoxic form have numerous applications in targeted cellular ablation. For example, the generation of cytotoxic metabolites that have low bystander potential (i.e., are largely confined to the activating cell) has been exploited for precise ablation of specific cell types in animal and cell-culture models; while enzyme-prodrug combinations that generate high levels of bystander cell killing are useful for anti-cancer strategies such as gene-directed enzyme-prodrug therapy (GDEPT). Despite receiving substantial attention for such applications, the canonical nitroreductase NfsB from *Escherichia coli* has flaws that limit its utility, in particular a low efficiency of conversion of most prodrugs. Here, we sought to engineer a superior broad-range nitroreductase, *E. coli* NfsA, for improved activity with three therapeutically-relevant prodrugs: the duocarmycin analogue nitro-CBI-DEI, the dinitrobenzamide aziridine CB1954 and the 5-nitroimidazole metronidazole. The former two prodrugs have applications in GDEPT, while the latter has been employed for targeted ablation experiments and as a precise ‘off-switch’ in GDEPT models to eliminate nitroreductase-expressing cells. Our lead engineered NfsA (variant 11_78, with the residue substitutions S41Y, L103M, K222E and R225A) generated reduced metabolites of CB1954 and nitro-CBI-DEI that exhibited high bystander efficiencies in both bacterial and 2D HEK-293 cell culture models, while no cell-to-cell transfer was evident for the reduced metronidazole metabolite. We showed that the high bystander efficiency for CB1954 could be attributed to near-exclusive generation of the 2-hydroxylamine reduction product, which has been shown in 3D cell culture to cause significantly greater bystander killing than the 4-hydroxylamine species that is also produced by NfsB. We similarly observed a high bystander effect for nitro-CBI-DEI in HCT-116 tumor spheroids in which only a small proportion of cells were expressing variant 11_78. Collectively, our data identify variant 11_78 as a broadly improved prodrug-activating nitroreductase that offers advantages for both targeted cellular ablation and suicide gene therapy applications.

## Introduction

**FIGURE 1:**
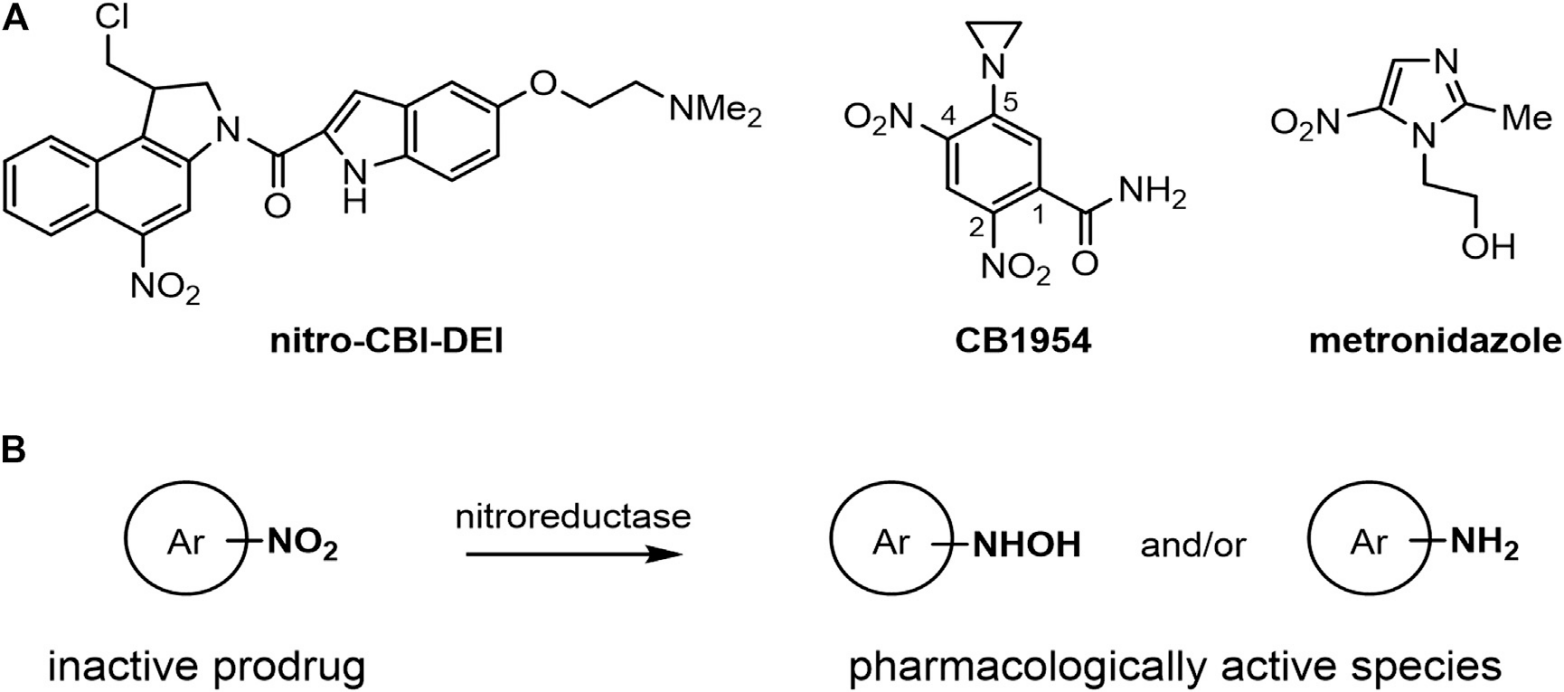
Structures and generic reaction scheme for the nitroaromatic prodrugs central to this work. **(A)** Structures of nitro-CBI-DEI, CB1954 and metronidazole. **(B)** Generic scheme showing nitroreductase-mediated activation of nitroaromatic prodrugs. The reduction of the strongly electron-withdrawing nitro group to electron-rich hydroxylamine or amine products can be used as a trigger to convert an inactive compound to products capable of alkylating DNA or proteins.

Bacterial nitroreductase enzymes are members of a diverse family of oxidoreductase enzymes that can catalyze the bioreductive activation of nitroaromatic compounds, including anti-cancer prodrugs such as nitro-CBI-DEI ((1-(chloromethyl)-5-nitro-1,2-dihydro-3*H*-benzo[*e*]indol-3-yl)(5-(2-(dimethylamino)ethoxy)-1*H*-indol-2-yl)methanone) and CB1954 (5-(aziridin-1-yl)-2,4-dinitrobenzamide), and antibiotic prodrugs such as metronidazole (2-methyl-5-nitroimidazole-1-ethanol) (Williams et al., 2015) (Figure 1). An important aspect of the toxicity of these prodrugs is their potential to exert a ‘bystander effect,’ i.e., the ability of their toxic metabolite(s) to exit the membrane of an activating cell and enter neighboring cells. Prodrugs with high bystander potential, such as nitro-CBI-DEI and CB1954, are of particular importance for suicide gene therapies where the vector fails to reach a majority of target cells, e.g., viral-directed enzyme prodrug therapy (VDEPT) (Dachs et al., 2009). Issues of heterogenous gene delivery can be mitigated through diffusion of the activated drug from transduced cells into non-transgenic and locally adjacent tissues. The bystander effect of the prodrug is even more critical for bacterial-directed enzyme prodrug therapies (BDEPT), where the activated prodrug must efficiently exit the bacterial vector cell to have any cytotoxic effect on tumor cells.

Conversely, prodrugs with low bystander potential such as metronidazole are well-suited for inducible and targeted ablation of specific nitroreductase-expressing cell populations within an organism. Transgenic animals that express a nitroreductase under control of a tissue-specific promoter have been used to study the physiological roles of cells during development, or to generate models of human degenerative disease in regenerative species such as the zebrafish (White and Mumm, 2013). Low-bystander prodrugs additionally have potential to provide an ‘off-switch’ for nitroreductase-based cellular therapies, enabling contained self-sterilization of nitroreductase-expressing vector cells. Such an ‘off-switch’ would improve the safety and controllability of gene therapy as it could be triggered at any required point upon administration of the low-bystander prodrug, and addresses specific concerns associated with the growing number of gene therapy technologies that are limited by a lack of therapeutic control following vector delivery (Goverdhana et al., 2005; Curtin et al., 2008; Das et al., 2016; Goswami et al., 2019).

Nitroreductases, and their potential applications, have been extensively studied over the past two decades. A particular appeal of using nitroreductases for cell targeting is the proven ability of many nitroaromatic prodrugs to kill quiescent as well as actively dividing cells (Denny, 2002). Additionally, the characteristic promiscuity of nitroreductases offers scope for developing a single enzyme to activate multiple useful prodrugs. However, the development of nitroreductase-mediated cell ablation tools or suicide gene therapies has been dominated by a single *E. coli* Type I nitroreductase, NfsB (Williams et al., 2015). This canonical nitroreductase exhibits only poor-to-moderate activity with nitroaromatic prodrugs such as metronidazole and CB 1954, requiring them to be administered at concentrations that approach toxic levels for any therapeutic effect to be achieved (Patel et al., 2009; Mathias et al., 2014). In addition, the bystander effect of CB1954 was lower than anticipated when assessed in multi-layer cell models expressing NfsB, likely because NfsB can reduce CB1954 at either the 2-nitro or the 4-nitro substituent, and reduction of the 4-nitro group generates a reactive metabolite that diffuses far less freely between adjacent mammalian cells (Helsby et al., 2004). In contrast, the alternative *E. coli* Type I nitroreductase NfsA is considered an excellent generalist, exhibiting higher activity than NfsB with a range of nitroaromatic substrates in both *in vivo* assays and *in vitro* purified protein kinetics (Vass et al., 2009; Prosser et al., 2013; Copp et al., 2017). Previous studies have also shown that NfsA reduces CB1954 solely at the 2-nitro position, exclusively generating the higher-bystander reactive metabolite (Vass et al., 2009; Prosser et al., 2010).

In this study, we aimed to engineer an improved version of *E. coli* NfsA exhibiting optimized activity with three prodrugs of particular interest: nitro-CBI-DEI, CB1954 and metronidazole. Our interest in the former two prodrugs was primarily for GDEPT applications, sparked by our observation that each is proficient at exiting *nfsA*-expressing *E. coli* cells and damaging co-cultured *nfsA*-null cells (Chan-Hyams et al., 2018). Metronidazole was also chosen for this study as its active metabolites are typically cell-entrapped post-reduction (Bridgewater et al., 1997), and it therefore has applications in precision cell ablation studies and as an off-switch to selectively eliminate gene therapy vector cells. To assess the suitability of our engineered enzyme for each intended application, we evaluated the activities of leading NfsA variants and the bystander properties of their activated prodrug metabolites in both bacterial models and human (2D and 3D) tumor cell models.

## Methods

### Materials, Bacterial Strains, Genes and Plasmids

All reagents were purchased from Sigma-Aldrich unless stated otherwise. CB1954 was purchased from MedKoo Biosciences, and nitro-CBI-DEI was synthesized in-house at the Auckland Cancer Society Research Centre, as previously described (Wilson et al., 2009). Nitroreductase candidates were over-expressed from the P_tac_ plasmid pUCX (Prosser et al., 2010) for bacterial assays, or pET28a(+) (Novagen) for expression and purification of protein. Bacterial growth assays employed the *E. coli* 7NT strain, which bears scarless in-frame deletions of seven candidate nitroreductase genes (*nfsA*, *nfsB*, *azoR*, *nemA*, *yieF*, *ycaK* and *mdaB*) and the efflux pump gene *tolC* (Copp et al., 2014). Expression of His_6_-tagged proteins was performed in *E. coli* BL21(DE3) cells (Novagen). The *nfsA* gene used in this study has the accession number WP_000189159.

### Library Construction

The *E. coli* NfsA 7RCM library was ordered as a synthetic gene cloned into the vector pUCX by GenScript, which guaranteed a total library size of 10 million unique variants. The lyophilized library was resuspended in Tris-EDTA buffer and was used either immediately in transformations or stored frozen at −20°C indefinitely. The *E. coli* NfsA 10SDM library was generated by introducing combinations of ten different individual amino acid substitutions (I5T, S41Y, E99G, L103M, K222E, R225A, R225G, R225P, F227S, L229V) that had been previously found to improve activation of the prodrug PR-104A (Copp et al., 2017).

### Bacterial IC_50_ Assays

Bacterial IC_50_ assays were performed as previously described (Prosser et al., 2013) with minor modifications as follows: (1) bacterial strains were cultured in LB medium supplemented with 100 μg mL^−1^ ampicillin and 50 µM IPTG; (2) day cultures (2 mL) were incubated for 2.5 h at 37°C, 200 rpm prior to transferal of thirty-two 30 µL-aliquots (allowing for testing of sixteen drug concentrations in duplicate) into wells of a 384-well microtiter plate containing 30 µL of ampicillin and IPTG-supplemented LB medium and a dilution series of metronidazole, CB1954 or nitro-CBI-DEI (including a no-prodrug control); (3) nitro-CBI-DEI challenge medium was additionally supplemented with 10 µM phenylalanine-arginine β-naphthylamide (PaβN) to inhibit TolC-mediated efflux (Green et al., 2013); (4) IC_50_ values were calculated using a dose-response inhibition four-parameter variable slope equation in GraphPad Prism 7.0 (GraphPad Software Inc)*.*

### Bacterial Bystander Effect Assays

Bacterial bystander effect assays were performed in 384-well microtiter plates as previously described (Chan-Hyams et al., 2018) with minor modifications as follow: (1) duplicate overnight cultures of a nitroreductase-expressing 7NT activator strain, a nitroreductase null 7NT control strain, and the SOS-R4 recipient strain were inoculated in 200 µL aliquots of LBASG media (lysogeny broth supplemented with 100 μg mL^−1^ ampicillin, 50 μg mL^−1^ spectinomycin and 0.2% glucose (w/v)) in a sterile 96-well microtiter plate and incubated overnight for 16 h at 30°C with shaking at 200 rpm; (2) the following morning, 0.3 mL of the overnight culture was used to inoculate a day culture of 5 mL LBASG supplemented with 50 µM IPTG, which was then incubated at 30°C, 200 rpm for 3 h; (3) four biological replicates were performed for each condition; each consisting of 3–6 technical replicates (biological replicates were derived from independent overnight cultures, technical replicates refer to separate experimental wells derived from the same overnight culture).

### HPLC Analysis of Reduced CB1954 Metabolites

Reactions of 100 µL containing 10 mM Tris-HCl pH 7.0, 1 mM NADPH and 200 µM CB1954 were initiated by addition of 1.75 µM purified enzyme (purified as His_6_-tagged proteins expressed from pET28a(+), as previously described; Prosser et al., 2013). Reactions were incubated for 25 min at room temperature before being stopped by addition of one volume ice-cold 100% methanol. Samples were transferred to −80°C for at least 1 h to precipitate proteins after which samples were centrifuged for 10 min at 12,000 *g*, 4°C. The supernatant was decanted and diluted 1:20 in 45 mM ammonium formate buffer (pH 6.5) containing 2.5% (v/v) methanol. A 100 µL volume of each sample was analyzed by reverse phase-HPLC employing an Agilent 1200 series system with an Ascentis C8 3 µm 150 × 4.6 mm column (Sigma-Aldrich). The mobile phase used for HPLC analysis was 45 mM ammonium formate buffer (pH 6.5) as aqueous and 80% acetonitrile as organic. The HPLC run parameters consisted of 4 min at 5% organic, a linear increase to 50% organic from 4 to 19 min and a further gradient increase to 70% organic from 19 to 21 min. The flow rate was 1.5 mL min^−1^ throughout and the eluate was monitored at 262 nm. Elution profiles from each nitroreductase-CB1954 reaction were compared against control reactions including *Bacillus subtilis* YfkO or *E. coli* NfsA which exclusively reduce CB1954 at the 4- or 2-nitro positions, respectively (Prosser et al., 2013).

### Creation of Nitroreductase-Expressing and Fluorescently Labeled Cell Lines

Genes encoding wild-type or variant NfsA proteins were PCR-amplified in Phusion™ PCR reactions (New England Biolabs) using oligonucleotide primers that introduced a mammalian Kozak consensus sequence, a Shine-Dalgarno prokaryote consensus sequence, a TAG stop codon and Invitrogen Gateway™ BP recombination sites (Supplementary Table S1). PCR fragments were recombined into the pDONR221™ vector (Invitrogen) using BP Clonase™ II enzyme mix (Thermo Fisher Scientific). Nitroreductase genes in the pDONR221™ vector were then recombined into the F279-V5 mammalian expression destination vector (Prosser et al., 2013) using LR Clonase™ II enzyme mix (Thermo Fisher Scientific). Plasmid encoding a nitroreductase (F279-V5:*ntr*), or a fluorescent protein (pCDNA3-GFP, Addgene #74165; or mCherry2-N1, Addgene #54517) was used to transfect HEK-293 cells at 70–90% confluency using Lipofectamine 3000 reagent (Thermo Fisher Scientific) as per the manufacturer’s instructions. To generate stable polyclonal cell lines, 48 h following transfection the media in transfected wells was exchanged for media supplemented with the appropriate selection antibiotic. Cells which had stably integrated plasmid DNA were selected for by multiple passage cycles in medium containing escalating concentrations of the selection antibiotic (1–3 µM in the case of puromycin, or 500–900 μg mL^−1^ in the case of G418) until cell death was no longer evident. Selection antibiotic concentrations were determined following generation of a dose response curve with nitroreductase-null cells. TagRFP657-expressing *nfsA*-null HCT-116 cells, with bi-allelic knockout of the *POR* gene encoding the endogenous one-electron oxidoreductase NADPH-cytochrome P450 reductase, were constructed as described previously (Hong et al., 2018). Briefly, an HCT-116 cell line in which both *POR* alleles were knocked out was transfected with the pTagRFP657-N1 plasmid (Addgene), which encodes TagRFP657 along with G418 resistance. Puromycin-resistant HCT-116 cells expressing variant 11_78 were generated as for the HEK-293 line, above.

### Western Blots

Nitroreductase expression in HEK-293 cell lines was assessed by Western blot. Cells were lysed in radioimmunoprecipitation assay (RIPA) buffer supplemented with 10 µL mL^−1^ protease inhibitor (P8340, Sigma-Aldrich). Samples were incubated on ice for 30 min, centrifuged at 17,000 *g*, 4°C for 20 min to pellet cellular debris, and protein in the supernatant was quantified using the BCA assay (Thermo Fisher Scientific) using BSA as a reference. Protein samples were separated by SDS-PAGE, transferred onto a PVDF membrane (Immobilon-FL, 0.45 μM pore size, Millipore) and blocked in TBS buffer containing 0.1% (v/v) Tween-20 and 5% (w/v) BSA. Blots were incubated for 4 h at 4°C with sheep anti-NfsA (a generous gift from Dr Peter Searle, University of Birmingham) diluted to 1:5,000 in TBS + 0.1% (v/v) Tween-20, or rabbit anti-α-tubulin (Abcam; ab18251) diluted to 1:5,000. Following incubation, the membrane was decanted and washed three times in TBS + 0.1% (v/v) Tween-20. For detection of the sheep anti-NfsA primary antibody, blots were incubated with a donkey anti-sheep IgG HRP-conjugated secondary antibody diluted 1:5,000 (Abcam, ab97125) prior to addition of SuperSignal^®^ West Pico Chemiluminescent Substrate (Thermo Fisher Scientific). Image acquisition was accomplished using a FujiFilm LAS-4000. For detection of the rabbit anti-α-tubulin primary antibody, blots were incubated with a goat anti-rabbit IgG Alexa 488-conjugated secondary antibody diluted 1:5,000 (Thermo Fisher Scientific, A-11008) and then visualized on a Fujifilm FLA 5100 fluorescence scanner.

### HEK-293 Cytotoxicity Assays

HEK-293 cell lines were seeded in 100 µL aliquots into 6.25 mm diameter culture wells at a density of 180,000 cells mL^−1^. Cells were seeded in RPMI medium supplemented with 1× glutaMAX, 10% fetal calf serum and 1% penicillin/streptomycin. Cells were left to adhere in a humified incubator at 37°C, 5% CO_2_ for approximately 16 h before being challenged with 100 µL of RPMI medium supplemented with 1× glutaMAX ± 2× the final metronidazole concentration. Cells were incubated at 37°C, 5% CO_2_ for 48 h. Following challenge, 10 µL of CellTiter 96 Aqueous One Solution Cell Proliferation Assay reagent (Promega) was added to each well (and also to a medium-only containing well to allow for baseline medium absorbance subtraction) and cells were incubated for 1 h further at 37°C, 5% CO_2_. Absorbance of wells was measured at 490 nm, and the absorbance value of the media-only control well was subtracted from all other measurements. The baseline-subtracted absorbance of challenged wells was compared to that of an unchallenged control well to determine percentage cell viability for each metronidazole concentration. IC_50_ values (the concentration of compound at which viability was reduced by 50% relative to the unchallenged control) were calculated using a dose-response inhibition four-parameter variable slope equation in GraphPad Prism 7.0 (GraphPad Software Inc). Calculated IC_50_ values are the averages of at least three biological replicates.

### Spheroid Bystander Experiments

Mono- and co-cultures of HCT-116:11_78 activator cells and HCT-116:*nfsA*-null target cells were seeded into Corning Costar 7007 ultra-low attachment round bottom plates (*In Vitro* Technologies) at 3,000 cells per well for 4 days. Cells were seeded in 20 µL of α-MEM (Thermo Fisher Scientific) supplemented with 10% fetal calf serum and 1% penicillin/streptomycin (Thermo Fisher Scientific). Plates were incubated overnight before adding a further 180 µL of media. On day four, spheroid size was estimated by microscopy (6 spheroids per seeding condition) and spheroid plates were transferred to an incubator chamber (BioSpherix Ltd.) connected to 95% O_2_/5% CO_2_ gas passing through a humidifier bottle. Plates were incubated for 30 min to remove central hypoxia and attenuate any subsequent metabolic activation of nitro-CBI-DEI by endogenous oxygen-sensitive one-electron reductases, before being exposed to increasing concentrations of nitro-CBI-DEI for 5 h. At the end of the incubation, spheroids were washed three times with PBS, and 80 µL of pre-warmed trypsin was added to each well. Four spheroids for each condition in duplicate were immediately pooled together into Titertube^®^ microtubes (Bio-Rad) containing a further 180 µL of trypsin. After a 5 min incubation at 37°C, these were pipetted at least 50 times for dissociation before adding 400 µL of media. Samples were mixed, and 10-fold dilutions performed. Each sample was counted using a Z2 Coulter particle counter (Beckman Coulter) to allow for accurate surviving fraction calculation. Cells were plated in 60 mm Petri dishes (*In Vitro* Technologies) at each cell density in either 2 µM puromycin (Thermo Fisher Scientific) or 1 μg mL^−1^ G418 (Thermo Fisher Scientific) media for selection of activator or target cells, respectively. Ten days post plating, plates were stained with methylene blue (Chem-Impex International Ltd.), and colonies with >50 cells were counted.

### Phenotyping Flow Cytometry

Spheroids and mono-layers (seeded in 6-well plates to be 80% confluent) were grown and exposed in parallel to 100 µM pimonidazole hydrochloride (Hypoxyprobe Inc) for 3 h under 95% O_2_ at 37°C to remove central hypoxia and attenuate metabolism of pimonidazole by oxygen-sensitive endogenous one-electron reductases. A total of 24 spheroids were pooled together, washed with PBS, and incubated with 500 µL pre-warmed trypsin. After 5 min incubation at 37°C, 500 µL of media was added and samples pipetted several times to aid dissociation. Monolayers were also trypsinized using 1 mL pre-warmed trypsin per well, followed by 1 mL of media. Cells were centrifuged at 1,000 rpm for 5 min, washed with PBS, and subsequently fixed with 1 mL of cold 4% methanol-free formaldehyde (Thermo Fisher Scientific). Samples were incubated in the fridge for 1 h, followed by adding PBS to dilute to 1% PFA and stored at 4°C until analysis. Samples were washed three times with cold PBS. They were then re-suspended in 1 mL of blocking solution consisting of PBS containing 0.1% Tween-20 (Thermo Fisher Scientific) and 1% BSA (MP Biomedicals NZ Ltd.) and incubated at 4°C for 30 min. Cells were centrifuged, blocking solution removed, and anti-pimonidazole antibody conjugated to FITC (Hypoxyprobe Inc) at 1:40 dilution in blocking solution was added to the samples and incubated overnight at 4°C. The next day, samples were washed three times (twice with PBS-Tween and once with PBS). Samples were analyzed using a BD Accuri C6 flow cytometer (BD Biosciences).

## Results

### Prodrug Activity Screening in *Escherichia coli* to Identify Improved NfsA Variants

To discover improved prodrug-converting nitroreductases we screened two previously-generated *nfsA* gene libraries: 1) a randomized codon mutagenesis library (“7RCM”) that uses NDT or NNK degenerate codons to randomize seven key residues in the active site of NfsA (S41, F42, F83, S224, R225, F227 and K222; Copp et al., 2020); and 2) a site-directed mutagenesis library (“10SDM”) that encodes all possible combinations of ten NfsA substitutions previously found to improve activation of the dinitrobenzamide mustard prodrug PR-104A (I5T, S41Y, E99G, L103M, K222E, R225A, R225G, R225P, F227S, L229V; Copp et al., 2017) (Supplementary Figure S1). Here we sought to probe both libraries for variants with an improved capacity to activate nitro-CBI-DEI, CB1954 and metronidazole. Each library was used to transform *E. coli* 7NT, a knockout strain that lacks seven endogenous nitroreductase genes as well as the *tolC* outer-membrane pump gene that governs efflux of several nitroaromatic prodrugs. Unlike the 10SDM library (512 possible variants), it was not feasible to directly screen the 7RCM library (∼96 million possible variants) in prodrug sensitivity assays. Instead, a pre-selection strategy was employed to remove 7RCM library clones comprising low- or non-functioning nitroreductase variants, or empty pUCX plasmid. Pre-selection involved plating transformed cells on agar plates supplemented with 0.5 or 5 µM niclosamide, a nitroaromatic antibiotic that is toxic to *E. coli:*Δ*tolC* cells but can be detoxified by functional nitroreductases (Copp et al., 2020).

As an initial selection for niclosamide detoxification has been shown to greatly enrich for NfsA variants that have an improved capacity to reduce metronidazole (Copp et al., 2020), we began by screening for improvement in this activity, followed by counter-screening to identify variants that were also improved with nitro-CBI-DEI and CB1954 (screening workflow summarized in Supplementary Figure S2). In total, 1750 10SDM library clones (a number predicted by GLUE (Firth and Patrick, 2008) to provide >95% library coverage) and 114 niclosamide pre-selected 7RCM library clones (57 from each niclosamide concentration) were screened for improved metronidazole activity via *E. coli* growth inhibition assays as previously described (Prosser et al., 2013). The top 20 metronidazole-activating gene variants were re-cloned into pUCX (to ensure no plasmid mutations) and used to transform fresh *E. coli* 7NT cells (to avoid resistance promoted by spontaneous chromosomal mutations), then counter-screened in IC_50_ assays with nitro-CBI-DEI and CB1954. From these assays, five nitroreductases from the 7RCM library (designated 5_4, 5_6, 5_14, 5_38, and 5_17) and four nitroreductases from the 10SDM library (11_78, 15_34, 12_54, and 11_9) were identified as exhibiting substantially improved activity with all three prodrugs (Table 1).

**TABLE 1:** IC_50_ values for *Escherichia coli* cells expressing the nine selected NfsA variants, wild type NfsA (NfsA WT), or pUCX control, following challenge with metronidazole (MTZ), CB1954 or nitro-CBI-DEI.

Variant	MTZ IC_50_ (µM)	CB1954 IC_50_ (µM)	Nitro-CBI-DEI IC_50_ (µM)	Library origin	AA substitutions
11_78	8 ± 3	90 ± 10	11.0 ± 0.5	10SDM	S41Y/L103M/K222E/R225A
12_54	8 ± 2	100 ± 10	11 ± 2	10SDM	R225A
5_4	8 ± 3	130 ± 30	11 ± 4	7RCM	S41R/F42Y/K222L/S224D/R225S
5_6	8.3 ± 0.8	110 ± 40	6.0 ± 0.1	7RCM	S41I/F83L/K222V/R225V/F227H
15_34	8.4 ± 0.7	70 ± 10	10 ± 1	10SDM	I5T/R225A/F227S
5_17	9 ± 3	120 ± 10	9 ± 1	7RCM	S41C/F42H/K222R/S224H/R225C/F227H
11_9	10 ± 1	100 ± 10	9.2 ± 0.3	10SDM	R225A/F227S
5_38	11 ± 2	90 ± 20	10 ± 3	7RCM	S41N/F42Y/F83L/K222R/S224R/R225N/F227H
5_14	11 ± 2	110 ± 6	10 ± 2	7RCM	S41N/F83V/K222T/S224R/R225N/F227S
NfsA WT	47 ± 6	220 ± 10	20 ± 3		
pUCX	>800	>1000	17 ± 2		

*Escherichia coli* 7NT cultures expressing either pUCX (empty vector control) or a nitroreductase were challenged for 4 h across serial dilutions of prodrug. Drug-dependent growth inhibition was measured by monitoring culture turbidity (OD_600_) pre- and post-challenge. Percentage growth relative to the unchallenged control was calculated for each drug concentration and these values were used to calculate prodrug IC_50_s. Data presented are the average of at least three biological replicates ± SD.

### Bacterial Bystander Effect Assays

**FIGURE 2:**
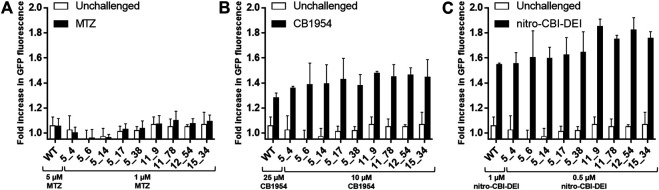
Microplate assay of SOS response induced by transfer of activated prodrug metabolites from nitroreductase-expressing 7NT bacterial activator cells to nitroreductase null SOS-R4 bacterial reporter cells. SOS-R4 cultures expressing GFP under the control of an SOS-responsive promoter, and 7NT cultures expressing an NfsA variant were mixed in a 50:50 ratio and then incubated with either 1 or 5 μM metronidazole (MTZ) **(A)**, 10 or 25 μM CB1954 **(B)**, or 0.5 or 1 μM nitro-CBI-DEI for 3.5 h **(C)**. Prodrug concentrations were varied between the wild type NfsA (“WT”) and variant NfsA expressing strains as indicated in each figure panel to ensure that the level of growth inhibition remained <20% (as we have previously shown that higher levels of growth inhibition can reduce output from SOS reporter gene assays; Prosser et al., 2010), with the higher concentration used for the wildtype NfsA-expressing strain, and the lower concentration for strains expressing one of the evolved variants. Cultures were analyzed for levels of GFP (ex 490 nm/em 510 nm) using a fluorescence microplate reader. For each prodrug and nitroreductase tested, fold-difference in GFP fluorescence was compared between a control condition (nitroreductase-null 7NT strain) and test condition (nitroreductase-expressing 7NT strain). Three biological replicates were performed, each consisting of eight technical replicates. Error bars represent standard deviation of the average fold increase in GFP induction across the three biological replicates.

To assess whether the engineered nitroreductases differed from native NfsA in their bystander profiles (e.g., by producing a different ratio of hydroxylamine to amine end-products for any of the prodrugs, or by losing selectivity for the 2-nitro substituent of CB1954), we first used a previously validated bacterial bystander model (Chan-Hyams et al., 2018) in which non-fluorescent ‘activator strains’ over-expressing an NfsA variant were co-cultured with a ‘reporter strain’ bearing an SOS-GFP DNA damage responsive gene construct. In all cases, the amino acid substitutions in the engineered NfsA variants were found to not affect their bystander profiles. Co-cultures incubated with metronidazole exhibited little to no GFP induction, reflecting sequestration of the activated metabolite(s) within cells of the non-fluorescent activator strain (Figure 2A). Conversely, co-cultures incubated with CB1954 or nitro-CBI-DEI exhibited strong GFP induction, indicating diffusion of the activated drugs from cells of the activator strain into cells of the reporter strain (Figures 2B,C).

### Evaluation of Evolved NfsA Variants in Human Cell Lines

**FIGURE 3:**
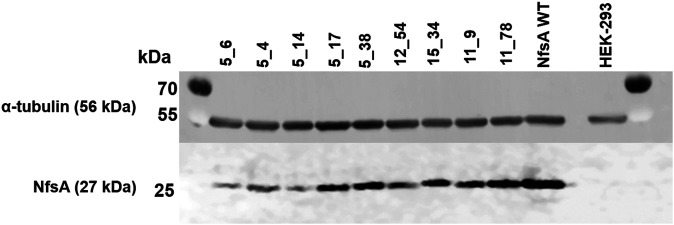
Western blot depicting expression of NfsA and variants by stably-transfected HEK-293 cell lines. HEK-293 cells were lysed with RIPA buffer and probed for NfsA expression by immunoblot using an NfsA polyclonal primary antibody and an anti-IgG HRP conjugate secondary antibody. The expression level of α-tubulin in samples was additionally determined via immunofluorescence detection and served as a loading control.

To explore whether the evolved NfsA variants might be suitable for VDEPT (which relies on the expression of a therapeutic enzyme by a transfected cell), or for targeted cell ablation studies, we assessed the abilities of each NfsA variant to sensitize a model human cell line (HEK-293) to the target prodrugs. Stable, polyclonal cell lines were generated that expressed either native NfsA or one of the nine variants. Expression testing via Western blot and subsequent densitometry analysis revealed varying levels of expression among variants in the stable cell lines (Figure 3). We have previously observed that even a single amino acid substitution can cause heterogeneous expression of NfsA variants in human cell lines or cause the expression of certain variants to be lost over time (Copp et al., 2017). While the reasons for this are as yet unproven, it may stem from the broad substrate promiscuity of NfsA (Valiauga et al., 2018), such that certain mutations confer the potential to interfere with essential metabolic pathways, and selecting for cells that have minimized or eliminated the expression of that variant. Based on their poor expression, variants 5_6, 5_4, and 5_14 were discontinued from further testing.

Cytotoxicity assays were then performed to compare prodrug activation by HEK-293 cells expressing either wild-type NfsA or one of the remaining variants. All variant-expressing cell lines demonstrated comparable or increased sensitivities to both metronidazole and nitro-CBI-DEI relative to cells expressing wild-type NfsA (Table 2). In addition, all variant lines demonstrated increased sensitivity to CB 1954. Overall, variant 11_78 appeared the most promising, as cells expressing this enzyme exhibited significantly increased sensitivities to all three prodrugs relative to cells expressing wild-type NfsA (*p* < 0.0001 for metronidazole and CB 1954, *p* = 0.022 for nitro-CBI-DEI; Student’s *t*-test) (Table 2).

**TABLE 2:** IC_50_ values for stably-transfected HEK-293 cell lines expressing one of the nine selected NfsA variants, or wild type NfsA, following challenge with metronidazole (MTZ), CB1954 or nitro-CBI-DEI.

	MTZ IC_50_ (µM)	CB1954 IC_50_ (nM)	Nitro-CBI-DEI IC50 (nM)
11_78	2 ± 1	90 ± 20	6 ± 4
11_9	12 ± 3	170 ± 80	13 ± 6
15_34	50 ± 20	200 ± 100	9 ± 4
5_17	53 ± 9	240 ± 50	46 ± 7
5_38	60 ± 20	350 ± 50	7 ± 5
12_54	70 ± 10	480 ± 80	9 ± 4
5_4	Not expressed	Not expressed	Not expressed
5_6	Not expressed	Not expressed	Not expressed
5_14	Not expressed	Not expressed	Not expressed
NfsA	70 ± 10	1400 ± 300	30 ± 10
HEK-293	>1,000	>10,000	1,300 ± 300

HEK-293 cells over-expressing wild type NfsA, or an evolved variant, were challenged for 48 h across serial dilutions of metronidazole, CB1954 or nitro-CBI-DEI followed by addition of MTS reagent and incubation for a further 2 h. Nitroreductase-null HEK-293 cells (“HEK-293”) were included in assays to assess the inherent cytotoxicity of each prodrug in the unreduced form. The absorbance of challenged wells at OD_490_ was measured using a plate reader and compared to that of unchallenged control wells. Percentage viability relative to the unchallenged control was calculated for each drug concentration and these values were used to calculate prodrug IC_50s_. Data presented are the average of at least three biological replicates ± SD.

**FIGURE 4:**
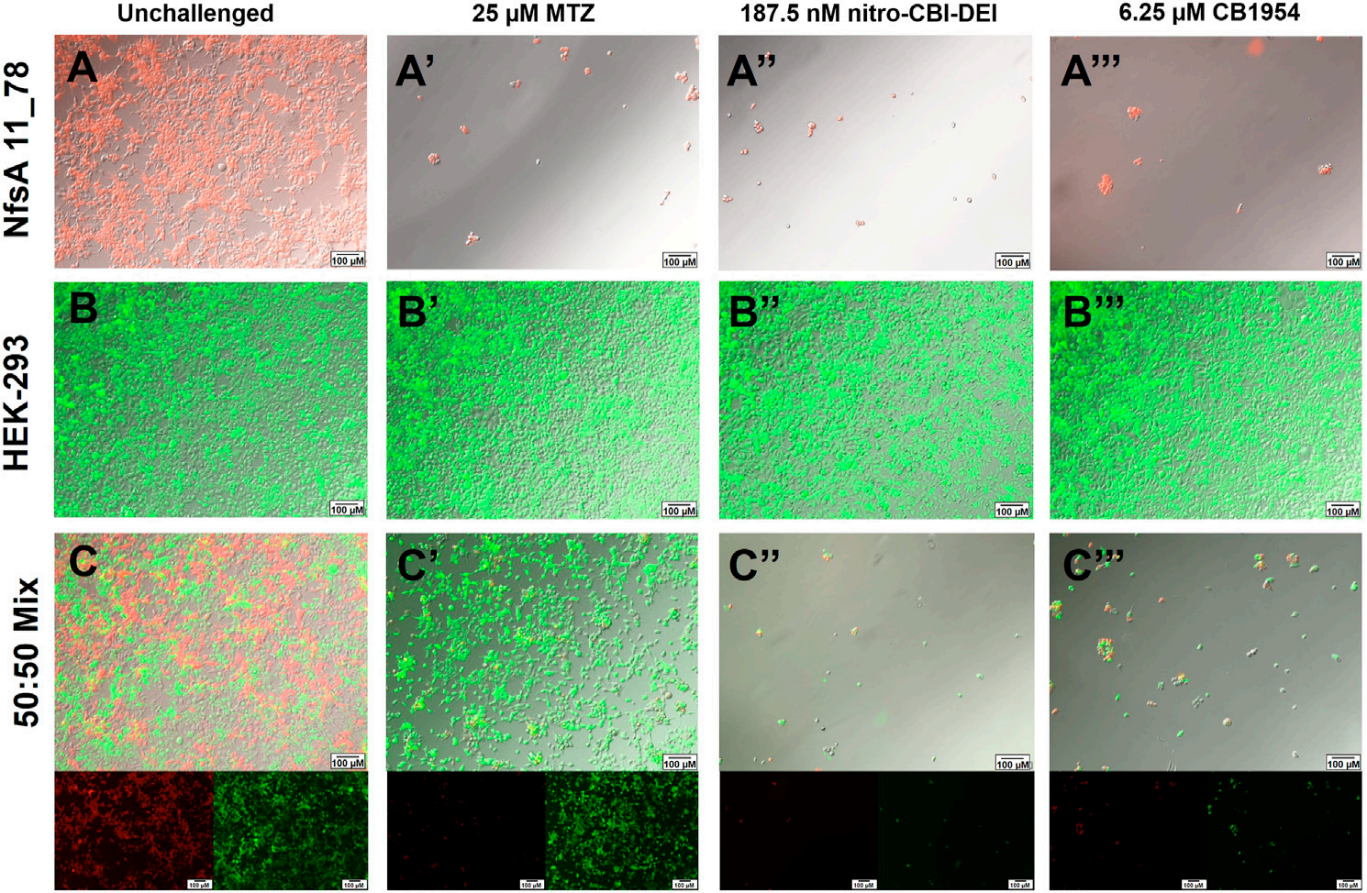
Ablation of mCherry-tagged HEK-293 cells expressing variant 11_78 (red) in co-culture with GFP-tagged nitroreductase-null HEK-293 cells (green) following metronidazole, nitro-CBI-DEI or CB1954 challenge. 11_78-expressing or nitroreductase-null HEK-293 cell lines were fluorescently tagged with mCherry or GFP respectively and grown in isolation **(A,B)** or in a 50:50 co-culture of HEK-293:gfp:HEK-293:mCherry+11_78 cells **(C)**. Cells were challenged with 25 µM metronidazole (MTZ) **(A′–C′)**, 187.5 nM nitro-CBI-DEI **(A″–C″)**, 6.25 µM CB1954 **(A‴–C‴)** or an equivalent volume of DMSO **(A–C)** for 48 h, after which cell viability was visualized by fluorescence microscopy.

We next sought to qualitatively assess cell-to-cell transfer of activated prodrug metabolites in the HEK-293 cell line expressing variant 11_78. For this, fluorescent HEK-293 cell lines were generated that stably expressed genes encoding either GFP alone, or mCherry and variant 11_78. Monocultures or co-cultures of the two cell lines were grown in monolayers and challenged with 25 µM metronidazole, 190 nM nitro-CBI-DEI or 6.3 µM CB1954 for 48 h. These prodrug concentrations were chosen from empirical tests as being 100% toxic to HEK-293:mCherry+11_78 cells (Figures 4A–A‴) and non-toxic to the nitroreductase null HEK-293:gfp cells (Figures 4B–B‴). Fluorescence imaging of the co-cultured cells challenged with metronidazole revealed targeted, cell-specific ablation of the nitroreductase-expressing cells (red), while nitroreductase-null cells (green) were unaffected (Figures 4C–C′’). In contrast, co-cultures challenged with nitro-CBI-DEI (Figure 4C″) or CB1954 (Figure 4C‴) showed complete cell death of both cell types, resulting from diffusion of the activated prodrug metabolites out of the nitroreductase-expressing activator cell, and into neighboring nitroreductase-null recipients.

### Evaluation of CB1954 Reduction Products

**FIGURE 5:**
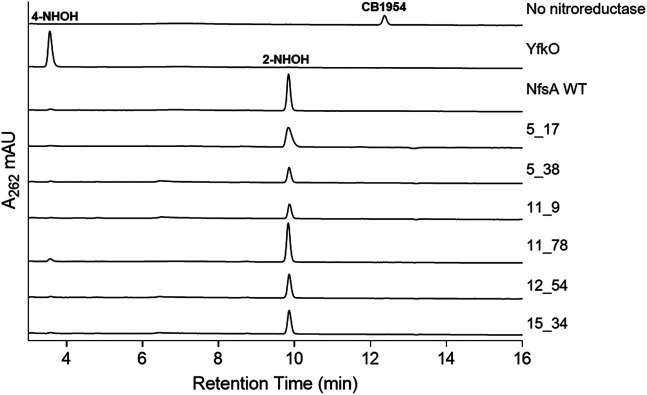
HPLC analysis of reaction products of CB1954 reduction by purified nitroreductases. Purified His_6_-tagged nitroreductases were incubated with CB1954 (200 µM) and NADPH (1 mM) in 10 mM Tris pH 7.0 for 25 min at room temperature prior to chromatographic separation of reaction end-products. Column eluates were monitored at 262 nm. Spectral peaks corresponding to the 4-hydroxylamine (4-NHOH) and 2-hydroxylamine (2-NHOH) end-products, or non-reduced CB1954, were determined by comparison with the YfkO, NfsA or no nitroreductase control traces, respectively.

To complement our 2D cell-based observations, CB1954 metabolite ratios were assessed at a purified protein level. Reduction of CB1954 by bacterial nitroreductases can potentially occur at either the 2-nitro or the 4-nitro functional group, but not both, to generate derivatives that elicit cytotoxicity through distinct mechanisms (Knox et al., 1991; Helsby et al., 2003; Helsby et al., 2004). Reduction at the 4-nitro position can generate a more potent cytotoxin capable of inter-strand DNA cross-linking, via an *N*-acetoxy intermediate (Knox et al., 1988), however reduction at the 2-nitro position yields metabolites that exhibit a substantially higher bystander effect in 3D cell cultures (Helsby et al., 2004). Given the importance of the bystander effect for GDEPT applications, we considered it desirable that none of the engineered NfsA variants had relaxed their exclusive specificity for the 2-nitro substituent. To assess this, the relative levels of 2-hydroxylamine and 4-hydroxylamine produced *in vitro* by the remaining NfsA variants were measured by reverse-phase HPLC. As standards, the end-products of CB1954 reduction by *E. coli* NfsA and *B. subtilis* YfkO were examined (these enzymes having been shown to exclusively generate the 2-hydroxylamine and 4-hydroxylamine end-products, respectively Vass et al., 2009; Prosser et al., 2013). All engineered NfsA variants were found to retain a strong preference for reduction at the 2-nitro position (Figure 5), i.e., yielding the end-products known to cause the highest levels of bystander cell killing in 3D tumor models (Helsby et al., 2004).

### Evaluation of Variant 11_78 and Nitro-CBI-DEI in Tumor Spheroids

**FIGURE 6:**
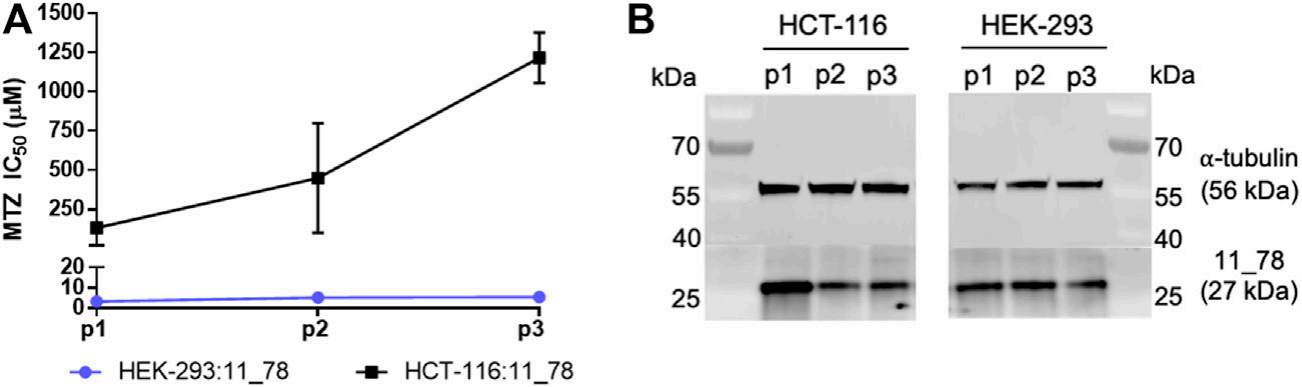
Metronidazole-sensitivity and 11_78 expression levels from HEK-293:11_78 and HCT-116:11_78 cell lines at three successive passages. At passage 1, 2, and 3 (days 7, 13, and 16 post-thaw, respectively), HEK-293:11_78 and HCT-116:11_78 cell lines were challenged with metronidazole (MTZ) (0–2,000 µM) for 48 h. Following drug incubation, cell viability at each metronidazole concentration was assessed by MTS assay and IC_50_ values were calculated. Data presented are the average of two technical replicates ± SD **(A)**. In parallel, samples of each cell line at passage 1, 2, and 3 were taken, lysed with RIPA buffer and probed for NfsA expression by immunoblot using an NfsA polyclonal primary antibody and an anti-IgG HRP conjugate secondary antibody. The expression level of α tubulin in samples was determined via immunofluorescence detection and served as a loading control **(B)**.

We finally sought to test whether the high bystander potential of nitro-CBI-DEI would also manifest in a 3D tumor model where only a minority of cells express NfsA variant 11_78. To enable generation of tumor spheroids we first created a stable polyclonal human colon carcinoma (HCT-116) cell line expressing variant 11_78. As a preliminary assay to confirm nitroreductase functionality, we tested sensitivity to metronidazole. We were surprised to observe in pilot tests of monolayer cultures that this cell line lost sensitivity to metronidazole over time (Figure 6A), suggesting that expression of variant 11_78 was not well-tolerated in these cells. Western blotting confirmed that the average amount of variant 11_78 protein per cell diminished as the passage number increased in HCT-116 cells, but not in HEK-293 (Figure 6B).

Although this phenomenon precluded us from establishing tumor spheroids that contained an *a priori* known ratio of nitroreductase-null cells to cells expressing variant 11_78, we reasoned that we could infer the ratio at the time of nitro-CBI-DEI challenge from replicate unchallenged spheroids by phenotyping them with α-[(2-nitro-1*H*-imidazol-1-yl)methyl]-1-piperidineethanol (pimonidazole). Pimonidazole has previously been shown to be reduced to a cell-entrapped form by *E. coli* NfsA and engineered variants thereof, enabling quantification of the proportion of NfsA-positive cells by flow cytometry (Copp et al., 2017).

**FIGURE 7:**
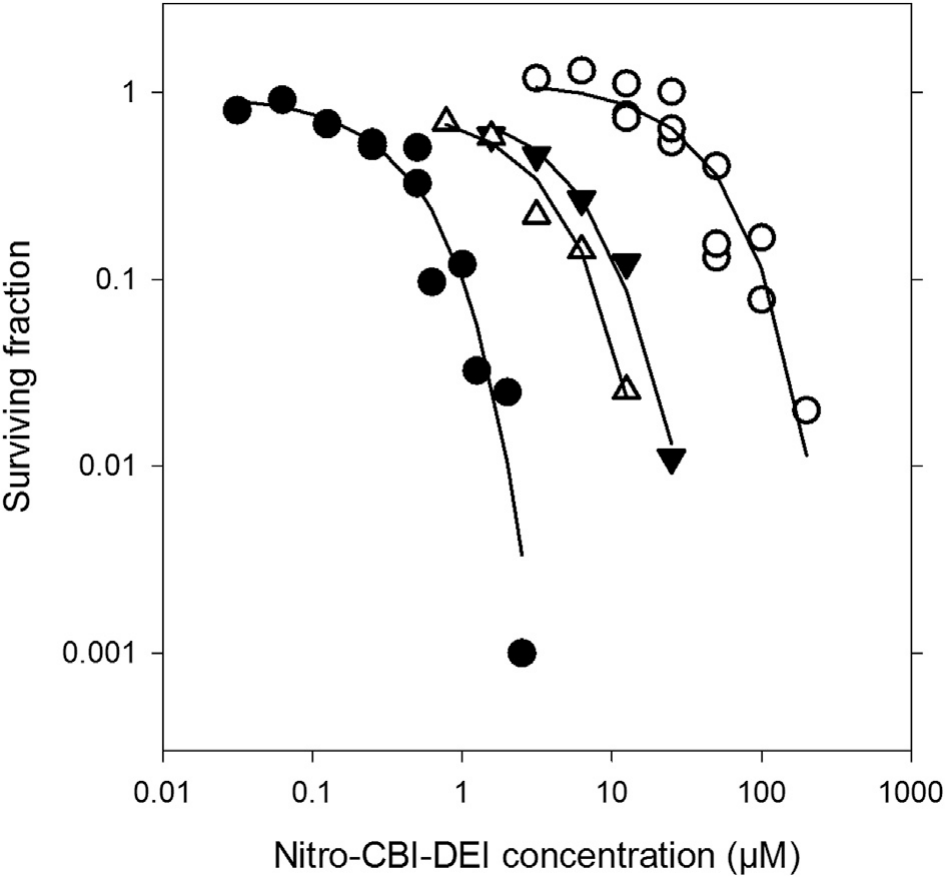
Clonogenic cell killing by nitro-CBI-DEI in spheroid co-cultures with HCT-116 *nfsA*-null and HCT-116:11_78 cells. HCT-116:11_78 and TagRFP657-expressing *nfsA*-null HCT-116 cell lines were grown in spheroid mono- or co-culture for 4 days. Spheroids were challenged with nitro-CBI-DEI for 5 h after which they were dissociated and plated in selective media to determine clonogenic cell survival. In parallel, untreated spheroids were phenotyped with pimonidazole and analyzed by flow cytometry (see Supplementary Figure S2). Closed circles: mono-cultures with HCT-116:11_78 cells; open circles: mono-cultures with HCT-116 *nfsA*-null cells; closed triangles: HCT-116 *nfsA*-null cells in co-culture with 10.5% pimonidazole-positive HCT-116:11_78 cells; open triangles: HCT-116 *nfsA*-null cells in co-culture with 33% pimonidazole-positive HCT-116:11_78 cells (phenotyping data not shown). Survival curves for each of the mono-cultures were from three pooled separate experiments.

For measurement of the nitro-CBI-DEI bystander effect, HCT-116 spheroids were grown from monocultures of either nitroreductase-null target cells or 11_78-expressing activator cells, or from co-cultures established at a varying starting ratios of nitroreductase-null to 11_78-expressing cells. Following 4 days of growth, mature spheroids (approximate diameter 540–640 µm) were exposed to increasing concentrations of nitro-CBI-DEI for 5 h. Simultaneously, unchallenged replicate co-cultured spheroids were dissociated to individual cells and phenotyped for nitroreductase activity using pimonidazole. Consistent with the cytotoxicity observed in monolayer cultures, nitroreductase-null spheroids were highly resistant to killing by nitro-CBI-DEI compared to monoculture spheroids expressing variant 11_78 (Figure 7). In co-culture spheroids, however, nitroreductase-null cells were markedly more sensitive to killing by nitro-CBI-DEI, resulting in a substantial left-shift of the survival curve. In two independent repeats, replicate co-culture spheroids were found to comprise 10.5% or 33% 11_78-expressing cells, with slightly greater sensitivity to nitro-CBI-DEI being experienced by the nitroreductase-null cells that were co-cultured with the higher proportion of 11_78-expressing cells. Collectively, these data indicate that the activated metabolite of nitro-CBI-DEI diffuses efficiently from the 11_78-expressing cells to kill neighboring nitroreductase-null cells even in a relatively high-cells density spheroid model (cell density ca. 4 × 10^8^ cells ml^−1^).

## Discussion

This work has identified the engineered NfsA variant 11_78, bearing the amino acid substitutions S41Y, L103M, K222E and R225A, as a promising nitroreductase for a broad range of cell-targeting applications. Not only was 11_78 the most generally-improved of the variants tested in this study (Table 1), on the basis of the IC_50_ values we measured in stably-transfected HEK-293 cells this is one of the most active nitroreductases to have been reported to date, with each of the three prodrugs. For example, the IC_50_ for this cell line with nitro-CBI-DEI is nearly 2-fold improved over HCT-116 cells expressing *E. coli* NfsB (Wilson et al., 2009; the only previous report of a human cell line IC_50_ for nitro-CBI-DEI), while the IC_50_ with CB1954 is comparable to those of the top five nitroreductase-expressing HCT-116 lines examined in a broad survey of 47 promising nitroreductase candidates (Prosser et al., 2013). Most notably, the IC_50_ with metronidazole is nearly three logs better than the IC_50_ we previously measured for *E. coli* NfsB expressed in the same HEK-293 cell line (Sharrock et al., 2020). *Escherichia coli* NfsB has been widely used in transgenic animal models, in particular zebrafish, to ablate promoter-defined cell types; however, these models have frequently been confounded by a requirement to apply near-toxic (or even toxic) doses of metronidazole to achieve effective cell ablation (Mathias et al., 2014). We recently addressed this limitation by developing “NTR 2.0” - a rationally engineered variant of *Vibrio vulnificus* that achieves effective cell ablation in transgenic zebrafish at 100-fold lower doses of metronidazole (Sharrock et al., 2020). In HEK-293 cells, variant 11_78 confers a 1.5-fold lower IC_50_ than even NTR 2.0, suggesting it will also be highly effective for targeted ablation applications. Variant 11_78 might therefore provide a useful alternative option for targeted cellular ablation, e.g., if NTR 2.0 proves not to be tolerated in certain models beyond zebrafish, provided 11_78 is expressed stably in those models.

We demonstrate here that variant 11_78 generates a low-to-no bystander cytotoxin from metronidazole, but high-bystander cytotoxins from CB1954 and nitro-CBI-DEI. It is possible that the latter two prodrugs might also prove useful in transgenic animal disease models, for example by exploiting their bystander effects to mimic traumatic or degenerative injury across a localized region. However, we envisage that the primary utility of these prodrugs will continue to be in anticancer (directed enzyme-prodrug therapy; DEPT) applications, where a strong bystander effect is often viewed as essential (Dachs et al., 2009). Our surprising observation that variant 11_78 is expressed stably in HEK-293, but lost from HCT-116 cells over successive passages, emphasizes that it will be important to test 11_78 in models that accurately reflect the intended form of DEPT. For example, BDEPT applications require that the prodrug-converting enzyme be stably expressed by the tumor-targeting bacterial strain, but do not have any requirement for human cell expression. In contrast, VDEPT scenarios do require that the therapeutic gene be expressed effectively in tumor cells, but are likely to reflect a transient rather than long-term expression scenario. Thus, assessing efficacy in transient viral transfection models is likely to be more meaningful than testing tumor xenografts derived from an initial mixture of 11_78-expressing and 11_78-null cells. Some other enzyme-delivery scenarios such as antibody-DEPT (Sharma and Bagshawe, 2017) or magnetic nanoparticle-DEPT (Anderson et al., 2019) may not require gene expression at all, whereas cellular therapies such as mesenchymal stem cell-based GDEPT (Amara et al., 2014) may have an absolute requirement for stable gene expression.

The preferred therapeutic prodrug for these different DEPT scenarios may also vary on a case-by-case basis, but in general we view nitro-CBI-DEI as a promising option based on its substantially superior dose-potency and heightened bystander effect relative to CB1954 (Williams et al., 2015). It is worth noting that the nitro-CBI-DEI tested here was a racemate, and moving forward it will be worth teasing apart the individual contributions of the *R* and *S* enantiomers, in case they differ in their cytotoxicity, bystander effect, or other key aspects. The ability of 11_78 to sensitize cells to metronidazole also provides an attractive off-switch for certain DEPT strategies (e.g., BDEPT, mesenchymal stem cell GDEPT) that could be triggered to kill nitroreductase-expressing cells at any point by administration of metronidazole. An important safety concern in suicide gene therapies is the risk of toxic side-effects that could arise from off-target localization of the gene therapy vector (Goverdhana et al., 2005; Curtin et al., 2008; Das et al., 2016; Goswami et al., 2019). A built-in metronidazole off-switch would thus provide a clean mechanism to safeguard against such adverse events, specifically ablating vector cells without harm to the host. As metronidazole is frequently given to cancer patients as an antibiotic (Zahar et al., 2005; Rosa et al., 2014), its dosing and potential side-effects are already well understood.

In summary, we envisage that the versatile prodrug-activation profile of this enzyme, improved in activity with each of three key prodrugs over the native generalist nitroreductase NfsA, make it ideally suited for both targeted cell ablation and DEPT therapies. We also anticipate that the new combinations of beneficial residue substitutions identified here within 11_78 and the other improved NfsA variants will contribute to a broader understanding of the phenotypic consequences of key substitutions, and may inform more rational approaches to engineering improved nitroreductase variants in the future.

## Data Availability

The raw data supporting the conclusions of this article will be made available by the authors, without undue reservation.
