# Supplementary material for: Engineering the Escherichia coli Nitroreductase NfsA to Create a Flexible Enzyme-Prodrug Activation System
Source: Front Pharmacol. 2021 Jun 7;12:701456. doi: 10.3389/fphar.2021.701456 (PMC8215503; doi:10.3389/fphar.2021.701456)
Supplement: Supplementary file 1 [file DataSheet1.docx]

**Supplementary Figure 1 – Structure of PR-104A**


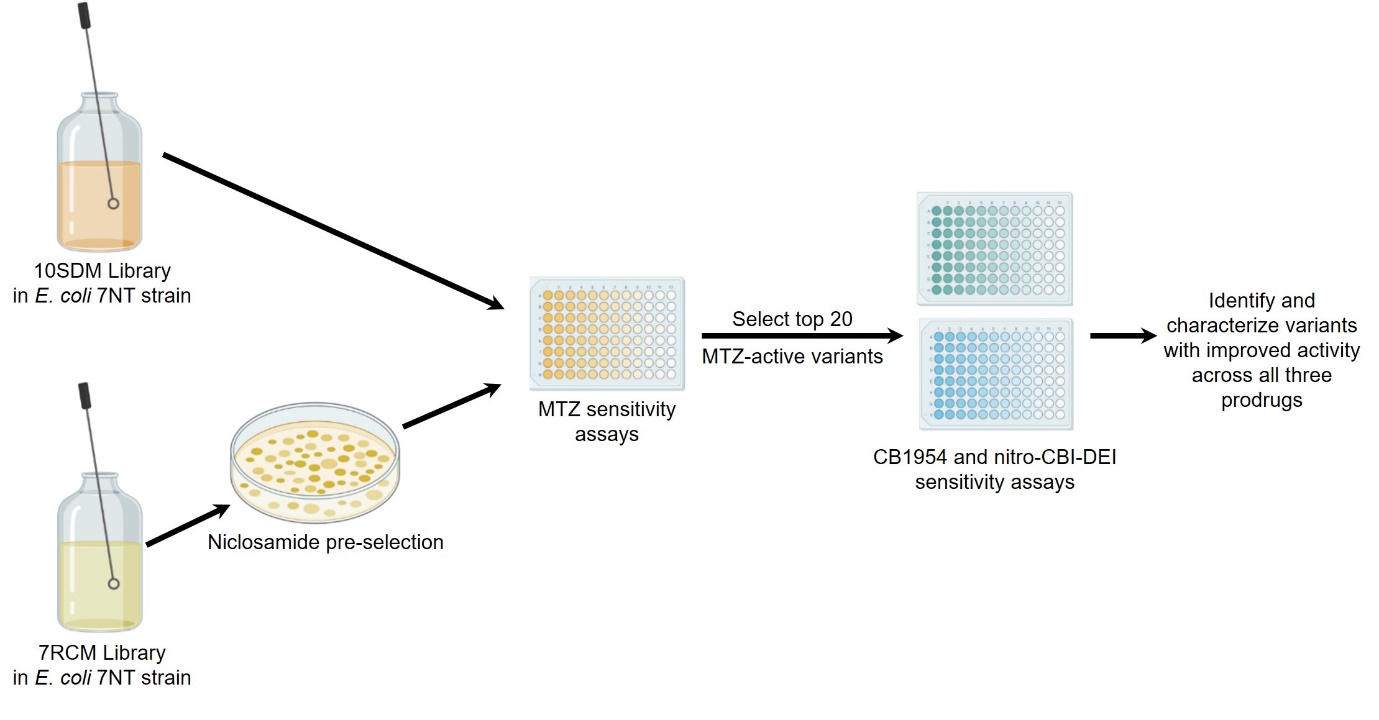


**Supplementary Figure 2 – Schematic summary of workflow for screening NfsA variants.** For the 10SDM library, 1750 clones were directly screened in *E. coli* growth inhibition assays to identify those most sensitive to metronidazole (MTZ). For the more extensively mutated 7RCM library, a niclosamide pre-selection was applied to enrich for active nitroreductases, after which 114 randomly-selected clones were screened for improved metronidazole activity. The top 20 metronidazole-active variants were then counter-screened to also quantify their activities with CB1954 and nitro-CBI-DEI.


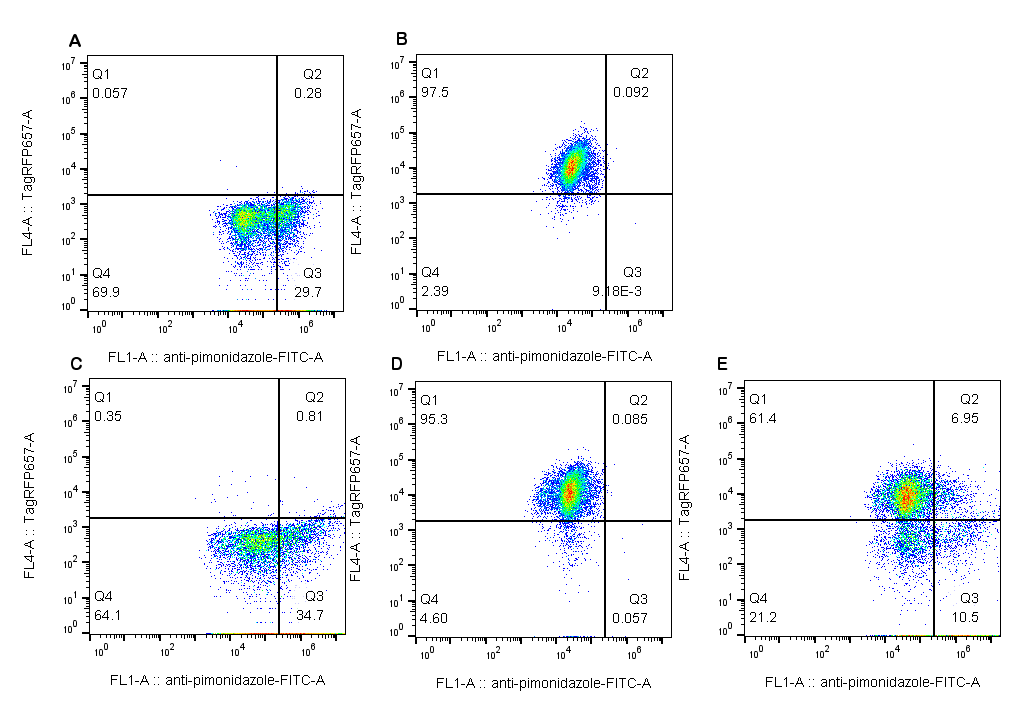


**Supplementary Figure 3 – Phenotyping of co-culture spheroids with HCT-116 cells expressing variant 11_78 and *nfsA*-null HCT-116 cells shown in Figure 7.** HCT-116:11_78 and TagRFP657-expressing *nfsA*-null HCT-116 cell lines were grown as monolayers (A, B) and in parallel spheroid mono- (C, D) or co-culture (E) for 4 days. Monolayers and spheroids were incubated with 100 µM pimonidazole for 3 h under 95% oxygen atmosphere, pooled (24 spheroids per condition), washed, and dissociated with trypsin into single-cell suspensions. Single cells were fixed in formaldehyde, incubated with FITC-conjugated anti-pimonidazole antibody, and analysed by flow cytometry. The cell suspensions from the monolayers were used as reference to set the gates. (A) HCT-116:11_78 cell monolayers, (B) TagRFP657-expressing *nfsA*-null HCT-116 cell monolayers, (C) HCT-116:11_78 cell mono-culture spheroids, (D) TagRFP657-expressing *nfsA*-null HCT-116 cell mono-culture spheroids, (E) spheroid co-cultures of HCT-116 *nfsA*-null cells and HCT-116:11_78 cells were found to comprise 10.5% pimonidazole-positive HCT-116:11_78 cells.

**Supplementary Table 1: Oligonucleotide Primers**

| Primer Name | Sequence (5’ – 3’) |
| --- | --- |
| NfsA Fwd | GGCATATGACGCCAACCATTGAAC |
| NfsA Rev | GGGTCGACTTAGCGCGTCGCCCAACCCTG |
| NfsA Gateway Fwd | GGGGACAAGTTTGTACAAAAAAGCAGGCTTCGAAGGAGATAGAACCATGGGCACGCCAACCATTGAACT |
| NfsA Gateway Rev | GGGGACCACTTTGTACAAGAAAGCTGGGTCCTAGCGCGTCGCCCAACCCTG |
